# Monthly Summary

**Published:** 1872-08

**Authors:** 


### MONTHLY SUMMARY.

*Disinfectants.*—A commission appointed by the French Academy to investigate the relative merits of various disinfectants for use in hospitals where contagious diseases are treated, have made the following report as the result of their experiments:

*Hyponitrous Acid.*—The members of the commission agree that the first place among agents for attacking and destroying infectious germs must be accorded to *hyponitrous* acid. Extraordinary precautions must, of course, be observed in making use of this dangerous gas; the doors and windows must be carefully sealed with gummed paper when disinfecting a room containing 40 or 50 cubic yards. The materials are taken in the following proportions: 2 quarts of water, 3¼ pounds of ordinary commercial nitric acid, and ½ pound of copper turnings or filings. A stoneware vessel is employed, holding two or three gallons. The exit doors are carefully pasted up, and the room left closed for 48 hours. The person opening the room at the expiration of the time should be protected in some way from breathing the gas, by a suitable respirator.

*Carbolic Acid.*—This is cheaper, more easily used, less dangerous, and has proved equally efficacious. It is best employed mixed with sand or sawdust—one pound of acid to three pounds of an indifferent substance. The mixture, placed in earthern vessels, was used for the same purpose as the hyponitrous acid. Carbolic acid, diluted with 15 or 20 parts by weight of water, was found useful for daily sprinkling of the floor and bed-clothes.

An interesting case is mentioned in the report where neither chlorine nor hydochlorous acid was able to destroy or render odorless the gases given off from the corpses in the Paris Morgue during the heat of summer. The object was attained by dissolving a quart of liquid carbolic acid in 500 gallons of fresh water, contained in the reservoir and used to sprinkle the bodies. Putrefaction was entirely stopped.

Devergie found that water containing only one to four thousandth part of its weight of carbolic acid sufficed to disinfect a dead house, even in the hottest weather, when six or eight corpses were in it.

For fumigating linen, mattresses and other bedding with chlorine, Régnault's latest method was used, namely: One pound of chlorinated lime (bleaching powder) is sewn up in a strong bag of sail cloth, holding about a quart, and put in an earthen pot containing a quart of common muriatic acid (sp. gr. 1.15) and three quarts of water. As soon as the acid comes in contact with the chloride of lime the room is closed, and things exposed to the action of chlorine gas for 24 hours; the room is then aired for 48 hours. Ten such earthen pots give off 500 litres of chlorine, sufficient to disinfect from 20 to 25, more or less, dirty mattresses.—*Scientific American.*

---

*Epithelioma Cured by Creasote.*—A noteworthy case is reported by Dr Forney, in the *Montpelier Medical*, of last February, showing the excellent effect of topical applications of creasote in epithelioma. A sailor, twenty-four years of age, presented himself, with an ulcer on the cutaneous border of the upper lip, about four centimetres in diameter. The tissues were perceptibly indurated in its vicinity, there was a severe itching pain, and there was no trace of syphilis. After two days' observation the ulcer was increasing, and was pronounced epithelioma.

Treatment was commenced by dipping a brush in pure creasote, and having removed any that might drop, the whole surface of the ulcer was lightly but firmly touched with a brush, a certain degree of pressure being used, and the brush retained on the same point a few seconds, so as to insure a thorough application. A piece of lint moistened in a gummy solution of creasote was then laid upon the ulcer. The application caused but slight pain.

Three days subsequently it was found that the ulcer had not increased. The application was repeated in the same manner, and five times afterwards at about the same interval. Cicatrization then commenced and proceeded rapidly, being favored by a dressing of thin paper moistened with a solution of creasote. Entire recovery took place about six weeks after the treatment was commenced.

This method deserves to be tried in similar cases.—*Medical and Surgical Reporter.*

---

*Physiological Action of Putty.*—A correspondent of the Philadelphia *Times* relates a case of a child who was troubled during October with constipation and acute strangury, making frequent attempts to void water, attended with much pain and screaming. There was no fever, the pulse was natural, tongue a little red, and the gums swollen from the imminent eruption of several teeth. The urine was "scanty, rather high-colored, and contained no albumen or casts, but a considerable number of very small oil-globules." These symptoms occurred at several different times, and were discovered, on careful investigation, to have been occasioned by handling and eating putty.

*The Blood in Syphilis.*—Dr. Lostorfer's now well-known alleged discovery of peculiar globules in the blood of syphilitic patients, was referred to a committee of the Vienna Academy of Medicine. While they were preparing to report, Prof. Wedl declared that "these so-called syphilitic corpuscles are nothing more than minute masses of fat and protoplasm, and are found quite as often in perfectly healthy as in syphilitic blood." The committee consequently reported that this closed the question, and further discussion was needless.

Poor Lostorfer must have been sadly disgusted at this exhibition of the *addictus jurare in verba magistri.* But an article by Prof. Biesladecki, in the *Wiener Medicinische Wochenschrift*, No. 8, 1872, will aid his cause a little. The Professor, who is of Krakow, assisted by his colleague, Prof. Stopczanski, found the corpuscles in the blood of persons with syphilis, rheumatism, heart disease, icterus, pneumonia, phthisis, variola, puerperal processes, septicaemia and heart disease. They are, he says, not fat, nor protoplasm, but paroglobulin granules; and they do appear more numerous in syphilitic than in other blood. They appear on the fourth or fifth day after the outbreak of an attack of syphilis, and on the seventh or eighth day in the other diseases mentioned.

In view of this we have not yet given up faith in Dr. Lostorfer and his corpuscles.—*Med. and Surg. Reporter.*

---

*A Simple Remedy for Dandruff.*—A writer in the *Journal of Pharmacy*, (*Boston Jour. of Pharmacy*), who had suffered much inconvenience from dandruff, and resorted to many advertised nostrums and other means for relief, among which were various alcoholic solutions of castor oil, and washing the scalp with solutions of borax and carbonate of potassa, which latter, although effectual for the relief from the dandruff, seemed to impair the vitality of the hair and cause it to become very sensibly thinner, was finally induced, from his knowledge of the frequent efficacy of sulphur in certain cutaneous affections, to try a preparation of an ounce of flowers of sulphur in a quart of water, as follows, with the happiest result: The sulphur was repeatedly agitated in the water during intervals of a few hours, and the clear liquid then poured off, with which the head was saturated every morning. In a few weeks every trace of dandruff had disappeared and the hair became soft and glossy, and now, after a discontinuance of the treatment for eighteen months, there is no return of the disease. Of the *modus operandi* of the treatment he offers no explanation—the effect speaks for itself.—*Medical Archives.*

